# Nanoscale patterning of a self-assembled monolayer by modification of the molecule–substrate bond

**DOI:** 10.3762/bjnano.5.28

**Published:** 2014-03-10

**Authors:** Cai Shen, Manfred Buck

**Affiliations:** 1 EaStCHEM School of Chemistry, University of St Andrews, St Andrews KY16 9ST, United Kingdom; 2 Ningbo Institute of Materials Technology & Engineering, Chinese Academy of Sciences, Ningbo, 315201, China

**Keywords:** copper, electrodeposition, gold adatoms, nanolithography, negative resist

## Abstract

The intercalation of Cu at the interface of a self-assembled monolayer (SAM) and a Au(111)/mica substrate by underpotential deposition (UPD) is studied as a means of high resolution patterning. A SAM of 2-(4'-methylbiphenyl-4-yl)ethanethiol (BP2) prepared in a structural phase that renders the Au substrate completely passive against Cu-UPD, is patterned by modification with the tip of a scanning tunneling microscope. The tip-induced defects act as nucleation sites for Cu-UPD. The lateral diffusion of the metal at the SAM–substrate interface and, thus, the pattern dimensions are controlled by the deposition time. Patterning down to the sub-20 nm range is demonstrated. The difference in strength between the S–Au and S–Cu bond is harnessed to develop the latent Cu-UPD image into a patterned binary SAM. Demonstrated by the exchange of BP2 by adamantanethiol (AdSH) this is accomplished by a sequence of reductive desorption of BP2 in Cu free areas followed by adsorption of AdSH. The appearance of Au adatom islands upon the thiol exchange suggests that the interfacial structures of BP2 and AdSH SAMs are different.

## Introduction

The applications of organic adsorbates for the electrodeposition of metals range from tuning the chemistry [1–2] to templating [3–4]. Contrasting the former where random assemblies are used, the latter relies on highly organised layers that comprise supramolecular networks [5–6] or self-assembled monolayers (SAMs) [3–4,7–18]. Exploiting variations in the interfacial charge transfer, SAMs are convenient systems to control the electrodeposition in a potential range both negative (overpotential deposition, OPD) and positive (underpotential deposition [19], UPD) of the Nernst potential. For the more common OPD, SAMs patterned by, for example, e-beam lithography [3,9], electrochemical printing [17], or colloidal masks [18] enable the selective deposition of metal structures and even their transfer to other substrates [4,12].

In contrast, UPD on SAM-modified electrodes yields a mono- or bilayer of metal, which is intercalated at the SAM–substrate interface [20–24]. The interest in this process arises from the alteration in the strength of the S–substrate bond. Following the order Au < Ag < Cu [25] patterning is enabled by a localised UPD of Cu or Ag on Au and the subsequent reductive desorption of the less tightly bound thiol molecules in the UPD-free Au areas to yield either nanoporous SAMs or binary SAMs in the case of backfilling with a second type of thiol [11]. So far, however, this approach has been lacking control as UPD is mediated by random defects [24,26–27] which, using standard SAMs such as alkanethiols, results in the arrangement of pores or domains of different thiols in a statistical fashion, thus, prohibiting patterning and controlling dimensions.

**Figure 1:**
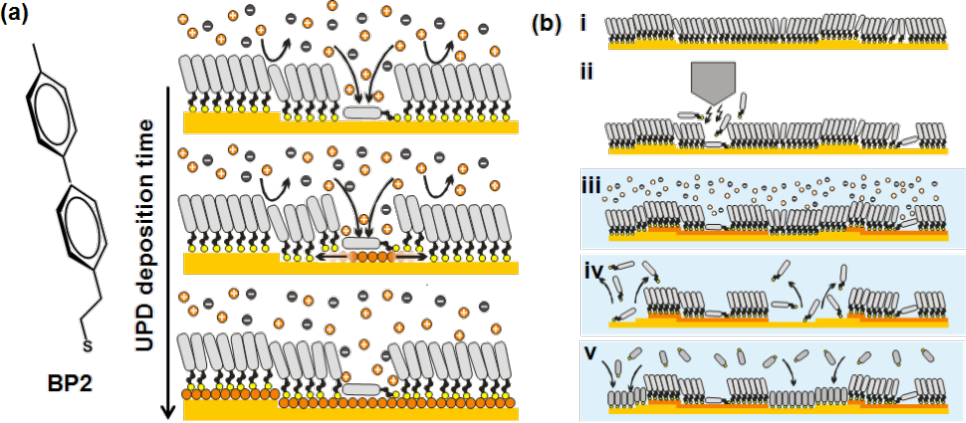
(a) Mechanism of Cu-UPD onto a BP2-modified Au(111) surface with the deposition starting at defects and the UPD proceeding by lateral diffusion of the metal atoms at the SAM–substrate interface. (b) Scheme illustrating the steps in UPD-based patterning. For details see text.

In order to overcome this bottleneck, SAMs are required that exhibit a structural perfection to an extent that UPD does not occur in the case of the native layer but only at defects introduced a posteriori by using lithographic techniques. In previous studies of our group it was found that SAMs of ω-(4'-methylbiphenyl-4-yl)alkanethiols (CH_3_-C_6_H_4_-C_6_H_4_-(CH_2_)*_n_*SH, BP*n*) can form layers of exceptional structural perfection [24,28–30], as a consequence of the specific molecular architecture characterised by an aromatic moiety linked to the thiol head group by a short aliphatic chain (see Figure 1a). Designing the molecules such that different factors that determine the enthalpy of the system compete to some extent [28], these SAMs can undergo phase transitions to structures that exhibit the required blocking of UPD. On Au substrates this is the case if the aliphatic spacer chain consists of an even number of methylene units. Two properties of the BP*n* SAMs are decisive for a patterned UPD process. The first one is that imperfections intrinsic to these layers, i.e., defects that cannot be eliminated such as domain boundaries and atomic steps in the underlying substrate, do not impede the passivation of these SAMs against UPD. More substantial defects such as impurities already present on the substrate prior to SAM formation or explicit damaging of the SAM are required. The second one refers to the mechanism of UPD, which is illustrated in Figure 1a. Different from what has been reported for alkanethiols [22,27] the UPD process starts at defects and proceeds via lateral diffusion of the metal atoms at the SAM–substrate interface. Most importantly, the UPD metal is exclusively supplied through the defects, not only in the initial stages of the process but until the whole surface is covered [24]. A crucial feature of the process is that the intercalation of the metal does not affect the passivating properties of the SAM. It is this defect- and diffusion-controlled UPD mechanism that forms the basis for the work presented here as patterned deposition becomes possible by a localised break down of the passivation and control over dimensions of UPD patterns will be exerted through the deposition time and/or the size of defects introduced.

While a range of lithographic techniques involving photons [31], electrons [32], ions [33], or scanning probes [30,34–36] is available for the high-resolution modification of SAMs, the modification by a tip of a scanning tunneling probe was chosen for practical reasons as patterning and characterisation can be conveniently done by the same instrument without altering the experimental setup. This is crucial for enabling the studies presented here, because to find isolated sub-100 nm structures reproducibly would become too tedious otherwise. It is, however, noted that this restriction does not apply if one is not interested in mechanistic in situ studies.

The overall process is outlined in Figure 1b. Starting from a high quality SAM (i) defects are introduced (ii) under ambient conditions by applying voltage pulses to the tip [7–8,37–38]. Subsequently, the sample is exposed to the electrolyte that contains the metal ions and UPD is performed (iii). Since, as illustrated in Figure 1a, UPD proceeds via diffusion of the Cu atoms and the deposition rate increases with higher cathodic potentials, the lateral dimensions are determined by controlling deposition time and potential. The UPD-modified SAM can then be further processed by removing the first thiol and then backfill the empty areas by a second thiol (iv), thus, creating a patterned binary SAM (v). It is noted that steps iv/v can be conveniently performed in one setup by reductively desorbing the first thiol in the cathodic sweep of a voltammetric cycle and adsorb the other thiol during the anodic sweep.

## Results and Discussion

### Patterned UPD

**Figure 2:**
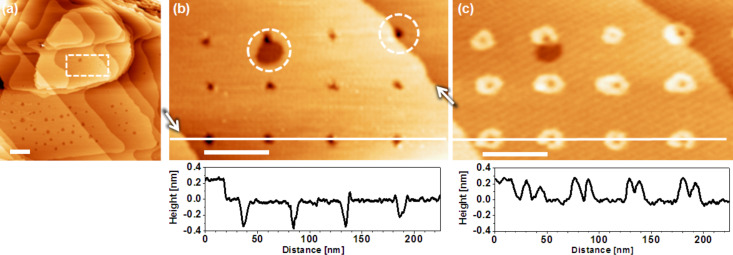
Cu-UPD on Au templated by a patterned BP2 SAM. a) Large scale STM image of the surface before deposition recorded in air. (b) Magnified image of area marked in (a) revealing an array of point defects created by voltage pulses of 4.5 V applied to the STM tip for 50 ms. Steps in the Au substrate are highlighted by the arrows. Dashed circles mark defects at the edge of a vacancy island and step, respectively. (c) In situ electrochemical STM image of the same area after Cu-UPD of 30 min at 0.275 V vs Cu^2+^/Cu. Height scales in the line profiles are normalised to the Au step height of 2.5 Å. All scale bars 50 nm.

STM images of the UPD of copper on a BP2-modified Au substrate are shown in Figure 2. The typical topography of the SAM-covered substrate is seen in Figure 2a. Due to the thermal treatment of the BP2 layer the smaller terraces are free of vacancy islands (VIs) and those present on more extended terraces are significantly bigger and less dense compared to samples prepared at room temperature. While Ostwald ripening accounts to some extent for this, the phase transition involved in the annealing is another process likely to contribute as discussed further below.

Defects in the SAM are introduced by pulsing the STM tip. The extent of damage depends on the voltage, and a value of 4.5 V was used in this example, which generates defects about 6 nm in size. As seen from Figure 2b the process yields pits of rather uniform size. A look at the line profile reveals that the depth of the depressions is typically 3–4 Å, which is somewhat larger than the 2.5 Å of the step height of the Au substrate. Taking previous studies into account [39] it is likely that thiols are removed together with gold atoms. Due to the lateral mobilitity thiols also diffuse from areas of the pristine SAM into modified regions. Therefore, the measured height changes are a superposition of topographical changes in the substrate and the SAM. After generation of the pattern in ambient environment the sample is exposed to the CuSO_4_ electrolyte and the UPD process is monitored in situ by electrochemical STM (EC-STM). According to the mechanism that is illustrated in Figure 1a [24] UPD starts at the defects and spreads radially. The EC-STM image of Figure 2c shows the surface after the growth of the Cu-UPD patterns for about 30 min at +0.275 V. After this period of time the circular UPD features have a diameter in the range of 12–20 nm. The rather anodic potential was applied to slow down the UPD process in order to allow in situ studies of the growth process. It is noted that if one is only interested in the generation of the UPD pattern the process can be significantly accelerated by depositing at more negative potentials or even extending into the OPD region. The features encircled in Figure 2b are interesting as they represent defects right at the edge of a VI and at a substrate step, respectively. They demonstrate that the presence of a step in the substrate does not affect the UPD process, i.e., even in close vicinity of the damaged SAM the passivation of the BP2 SAM across steps is not affected to the extent that the passivation against UPD breaks down. There is no UPD outside the damaged areas, which confirms the excellent quality of the BP2 SAM.

In experiments, in which we varied the spot size of the damage we noticed that this significantly affects the rate of the UPD process. In agreement with the mechanism established for this type of SAMs [24] this is expected since the growth rate scales with the flux of Cu ions integrated across the defect area. Interestingly, a minimum size of the defect was observed to be required. For defects smaller than 5 nm, it is difficult to trigger the UPD, or even if the UPD starts, the UPD can easily be blocked during the UPD process. This further corroborates that, after removal of thiols by pulsing, thiols diffuse back into the defect from the surrounding area. Obviously, the SAM can bear a certain level of disorder/defects before the passivation against UPD breaks down. Even though it was not a focus of the present study we note that the partial passivation of the defect by SAM molecules also requires substantially more cathodic potentials to initiate the deposition of bulk metal into these holes as compared to a clean Au substrate.

**Figure 3:**
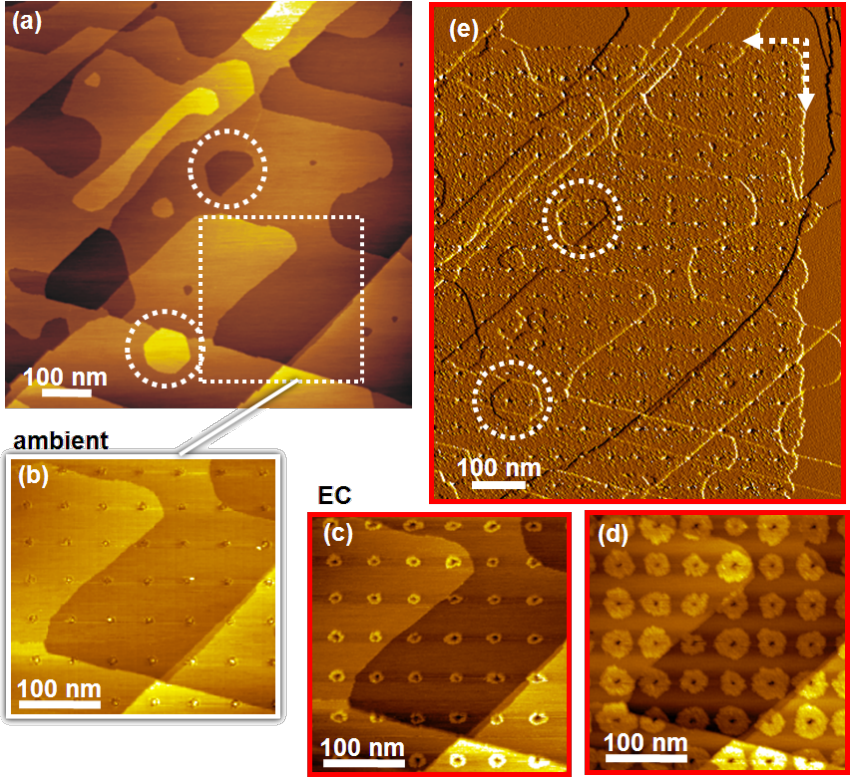
Temporal evolution of Cu-UPD. (a) Large scale ambient STM image of a native BP2 SAM on Au. (b) Magnified image of the area marked by the square in (a) after patterning with voltage pulses of 3.8 V for 50 ms. (c,d) EC-STM images of the area shown in (b) after different periods of UPD, 176 min (c) and 329 min (d) with the sample potential decreased from initially 0.4 V to 0.2 V vs Cu^2+^/Cu. (e) Large scale STM image after the UPD islands have coalesced to a uniform area. Circles in (a,e) mark identical areas. The dashed arrows in (e) mark boundary between UPD areas and passivated areas. (a–d) show constant current images, in (e) the derivative is shown for better visual differentiation between native and UPD modified areas.

An obvious feature of the UPD mechanism on BP2-modified substrates is to control the dimensions of the deposited metal through the deposition time. This is illustrated in the sequence of STM images depicted in Figure 3, which also illustrates the reproducibility of the process. After generation of the matrix of defects (Figure 3b) by using voltage pulses of 3.8 V/50 ms to yield defects in the range of 7 ± 3 nm, the continued deposition yields growing circular islands (Figure 3c,d) about 10–50 nm in diameter. Figure 3c shows a pattern of Cu-UPD that was formed after 176 min by progressively changing the sample potential from +0.4 V to +0.2 V during this period of time. All UPD islands exhibit a circular shape, with small contour variations at the edges. As the deposition continues the UPD islands grow as evidenced by Figure 3d, which shows the pattern formed after 329 min. The islands are about 30–50 nm in size. Their circular shape is still maintained, which demonstrates that these Cu patterns were formed by the Cu^2+^ ions diffusing radially out from the defects initially created by the STM tip. Ultimately the island coalesce to form a uniform UPD area (Figure 3e), which in the example displayed was accomplished after 486 min at 200 mV. In order to make the uniform deposition more easily visible, the derivative of the current is displayed in Figure 3e. The boundaries between the UPD and unmodified areas are marked by the dashed arrows and the features marked by the dashed circles provide the reference to the large scale image acquired in air prior to UPD.

**Figure 4:**
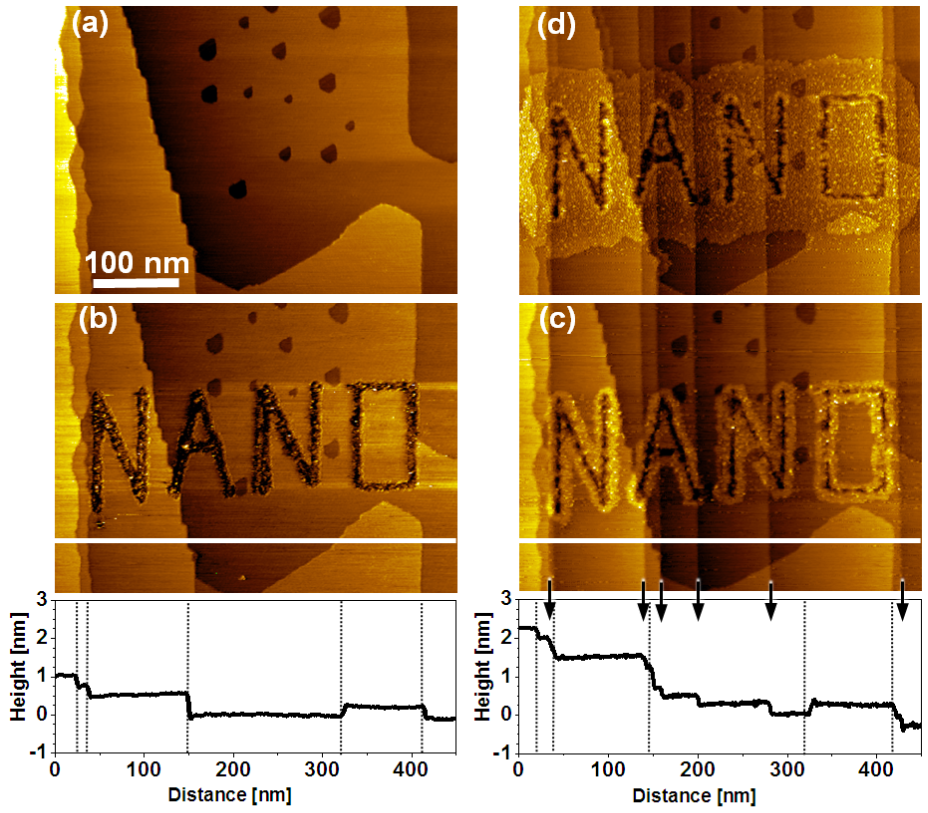
Templated Cu-UPD illustrating tolerance of the process against substrate dislocations. (a) Native substrate with uniform BP2 SAM. (b) Lithographic pattern formed in air by using a tip bias of 4.2 V and a tip speed of 0.75 μm/s. (c) EC-STM images of Cu-UPD after 11 min at 0.16 V vs Cu^2+^/Cu. (d) Uniform UPD area after 32 min. Dotted lines and arrows in height profiles along lines shown in (b,c) mark substrate steps present in the native substrate and generated during UPD, respectively.

The procedure is not limited to point like defects as illustrated by Figure 4. By using a bias of 4.2 V and a tip speed of 0.75 μm/s continuous lines such as the letters are written. As for the matrix of point defects, the UPD progresses until areas merge (Figure 4d). A salient feature of this example is the appearance of additional steps during the metal deposition, which is highlighted by the height profiles along the lines shown in Figure 4b and 4c and reflected by an integral step height of 1 nm and 2.5 nm prior and during deposition, respectively. Marked by arrows in the line profile of Figure 4c, the six additional steps that emerge during the electrodeposition are identical in height to the 2.5 Å of the Au steps present on the native substrate, thus, strongly suggesting that the Cu-UPD gives rise to dislocations in the Au surface. The tensile stress introduced by the Cu-UPD [40–41] adds to the stress already present in the substrate as a result of the preparation process and of defects in the mica substrate [42]. Obviously, the additional stress introduced by the UPD of Cu exceeds the threshold required to trigger a substrate relaxation by generating steps. As it can clearly be seen from Figure 4c and Figure 4d there is neither a penetration of UPD metal at newly created steps nor a preferential diffusion of UPD metal along those steps. Thus, possible structural differences between a BP2 SAM that covers a native step in the initial preparation procedure and one being generated during the UPD process are too small to alter the UPD mechanism. This is essential for the exploitation of this process on the nanoscale as the UPD pattern and, thus, its spatial resolution is not impeded by processes that cannot be eliminated.

The ruggedness of the BP2 SAM structure against the generation of Au steps, which is induced by the UPD, is in line with the preserved passivation of the monolayer at steps of Cu-UPD islands intercalated at the SAM–substrate interface [24]. However, the distinct generation of Au steps in the example presented above suggests that the STM patterning itself has an influence. While for a small point-shaped damage dislocations in the substrate occur rarely (none in Figure 2, one in Figure 3 intersecting the encircled island in the lower half of image (e)) the more extensive damage of the SAM by writing continuous lines (here in the form of letters) gives rise to a substantial number of substrate dislocations. This can be rationalized by considering that at least the topmost Au layer is affected, which includes the removal of Au atoms together with thiol molecules.

### Conversion of UPD pattern into binary SAM structure

The SAM modified by the UPD pattern corresponds to a latent image, which has to be developed by, for example, conversion into a pattern that exhibits heterogeneous surface properties as illustrated in Figure 1b. As mentioned above this is conveniently done by exploiting the differences in the strength of the S–metal bond between Au and Ag and Cu [25]. While the selective removal of thiols from UPD-free Au areas has been exploited for the generation of nanoporous SAMs [11], the process lacked control as UPD occurred at defects present in the native monolayer. The approach based on the SAMs used here, which perfectly block UPD, allows for the exploitation of this principle for the controlled patterning on the nanoscale.

**Figure 5:**
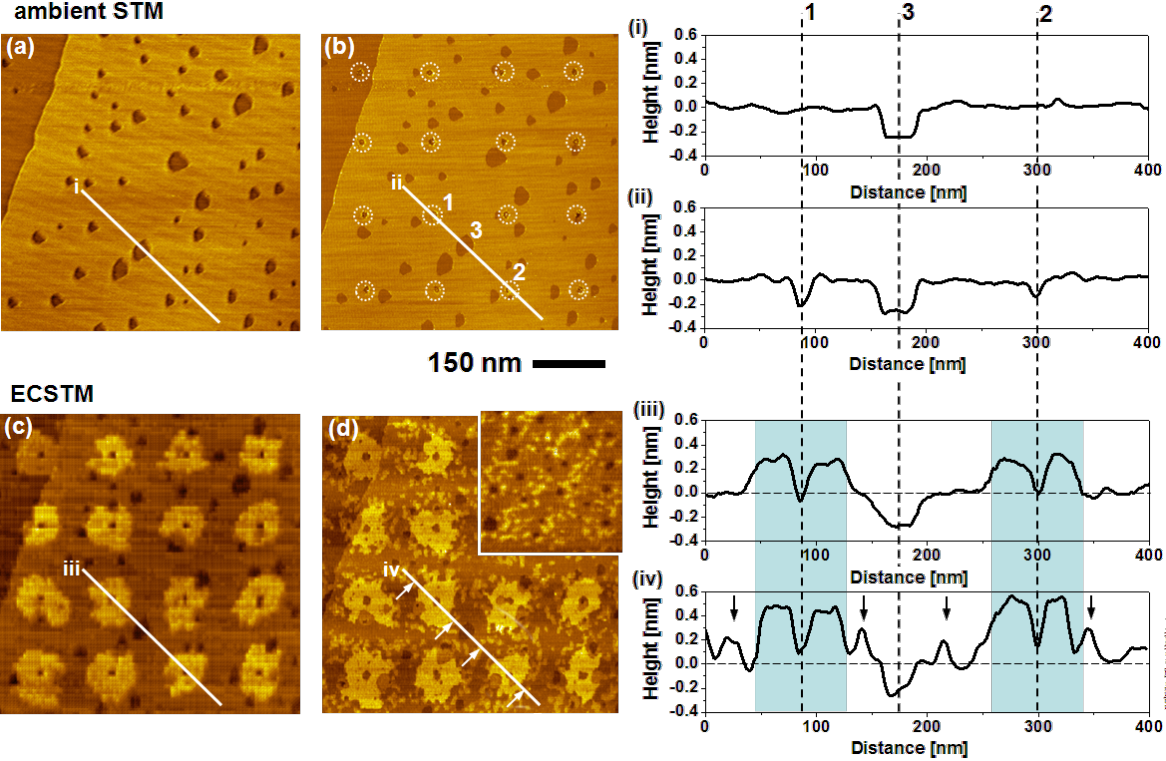
Sequence of STM images showing the UPD-based conversion of a BP2 SAM into a patterned binary SAM of BP2 and AdSH. Left: (a) Native BP2 SAM. b) Array of defects (encircled) created by STM lithography while using voltage pulses of 3.5 V and 50 ms duration. (c) Pattern of Cu-UPD generated by holding the sample potential at +0.3 V vs Cu^2+^/Cu for 10 seconds. (d) Binary SAM structure after reductive desorption of BP2 and adsorption of AdSH. The inset shows an area of the sample, which had not been modified by Cu-UPD. Right: Compilation of height profiles along lines shown in the STM images illustrating the evolution of topography. Protrusions marked by arrows in (iv) reflect AdSH covered Au islands. For details see text.

The concept is demonstrated in Figure 5, which shows a series of STM images that comprise the native (a), STM patterned (b), and UPD modified BP2 SAM (c), as well as the binary SAM (d), where BP2 adsorbed on Au has been replaced by adamantanethiol (AdSH). The exchange was accomplished by performing a voltammetric cycle, in which the reductive desorption of BP2 and the adsorption of AdSH occured during the cathodic and anodic sweeps, respectively. In the present experiment a basic solution of AdSH in EtOH was used. The successful exchange of the thiol is probed by a second cyclic voltammogram. An anodic shift of the desorption potential by about 35–40 mV (see Figure S1 in Supporting Information File 1) is characteristic for the difference in stability between the two thiols [43]. The exchange is also evidenced by characteristic differences seen in the STM images recorded before (Figure 5c) and after (Figure 5d) the replacement of BP2 in the areas, which were not covered by UPD islands. The most obvious one is that the contours of the islands become rather ill-defined and protrusions appear in between the UPD islands. While, at first glance, this seems like a serious deterioration of the shape of the islands, a closer look reveals that the contours of the islands such as shape asymmetries and irregularities are rather well preserved. The topographical changes are mainly due to the restructuring of the Au surface upon desorption of BP2. The fact that exactly the same topographical changes occur in areas of the sample where the SAM has not been patterned (inset in Figure 5d) proves that these features are not related to Cu-UPD. The formation of the protrusions agrees well with other studies of thiol desorption [44–45] and is explained by the formation of Au islands from Au adatoms present at the SAM–Au interface [46–48]. There is, however, a difference between the present study and other studies, in which island formation has been observed. Any Au islands formed during the desorption of BP2 should be consumed again when the other thiol is adsorbed. The extent to which this occurs is dependent on how similar the structures of the SAM–substrate interface are for the two thiols. Since the adamantanethiol packs less dense compared to BP2 (≈40 Å^2^ per molecule compared to ≈29 Å^2^) it is expected that Au islands remain after the adsorption of the adamantanethiol. However, the integrated area covered by the islands is unexpectedly large. Assuming that the number of Au adatoms involved is identical for BP2 and AdSH and that the same bonding configuration discussed for alkanethiols is adopted involving either one Au adatom per molecule or shared between two thiols, the area covered by islands should be about 3.5–7.0% of a monolayer after the exchange. This is significantly smaller than the experimentally observed area covered by islands, which amounts to at least 20%. It is noted that this rough estimation assumes i) a full monolayer of AdSH, ii) a packing density of atoms in the islands equal to that of bulk Au, and iii) a negligible effect of the tip shape on the measured island area. While a full monolayer might not have been formed (see CV) the coverage is not that low that it can account for the difference in numbers. Even though this conclusion is tentative and has to be backed by a separate, more detailed study it raises the question to what extent the structures of the SAM–substrate interface discussed for alkane thiols are realised in thiol SAMs whose packing densities are rather different. It is noted at this point that it has been argued that the pronounced phase transitions observed in BP*n* SAMs with *n* = even are hard to understand without a substantial restructuring of the SAM–Au interface [28–29].

The exchange of BP2 by AdSH is also reflected by a change in the relative height of the UPD islands. For the sample uniformly covered by BP2 (profile iii in Figure 5) the islands exhibit a height of 2.5–3.0 Å, which is in agreement with previous studies for this system [24]. After replacement the height has increased to 4–5 Å (profile iv), which is expected considering the smaller size of AdSH compared to BP2 and the aliphatic nature compared to the aromatic system.

## Conclusion

Thiol SAMs based on a molecular architecture, which combines structure determining factors in a competing manner [28], can be prepared in a polymorph, in which defects are eliminated to the extent that a gold electrode is completely passivated against UPD of Cu. This introduces new opportunities for the structuring of SAM on the nanoscale, as the deposition of copper is not determined anymore by randomly distributed defects that are usually present in a native SAM [11]. Instead, patterns of Cu-UPD can be freely defined by generating defects in a controlled fashion. Additional degrees of freedom are provided by the rate of the Cu deposition, which is determined by the size of the defects, and the deposition time, through which the extent of lateral diffusion of Cu at the SAM-substrate interface and, thus, the size of features is defined. In contrast to other patterning schemes, in which the final structure is a replica of the lithographic pattern, this allows to enlarge features and, thus, reduce the effort in the lithographic step, which is of advantage in high resolution patterning that use serial tip or beam based techniques.

The local modification of the sulfur–substrate bond by intercalation of Cu at the Au–substrate interface yields a latent image, which is straightforwardly developed into a patterned binary SAM. Harnessing the significant difference in the strength of the S–Au and S–Cu bond this involves a potential-controlled reductive desorption of the thiol in areas that are not modified by Cu-UPD followed by the adsorption of a second thiol. As such it is a negative resist technique and, thus, complementary to other lithography based schemes such as grafting [35], in which the replacement takes place in the written areas.

While patterning on a scale down to less than 20 nm has been demonstrated it has to be seen how far this patterning scheme can be extended towards the bottom end of the nanoscale. The factors that limit resolution and accuracy at present are related to the precision, at which defects in the SAM can be made and how well the diffusion of both the thiols and the intercalated Cu can be controlled. The use of, for example, an ion beam for SAM patterning instead of the voltage induced generation of defects is anticipated to further improve the accuracy and reproducibility of the Cu-UPD. The timing in the thiol substitution is another parameter to be optimised in order to minimise the blurring of contours by the diffusion of species. While UPD-based patterning has been demonstrated here for the generation of a binary SAM the scope of this scheme reaches further. In particular, the contrast in charge-transfer properties between the passivating UPD-modified SAM islands and the active electrode areas, which are generated by reductive desorption of thiols, makes the scheme attractive for electrodeposition on the nanoscale. An extension to other metals, which include catalytically active or magnetic metals deposited at both underpotential and overpotential, or to semiconductors makes the present scheme interesting for the generation of functional nanostructures. Furthermore, a deposition in the overpotential range at the defects offers the possibility to generate well-defined arrays of metal clusters provided the size of the defects in the SAM can be precisely controlled.

## Experimental

**SAM preparation.** 2-(4'-Methylbiphenyl-4-yl)ethanethiol (BP2) was synthesized as described previously [49]. Adamantanethiol (Sigma-Aldrich) and absolute ethanol (BDH) were used as received. Substrates (300 nm Au film on mica) were purchased from Georg Albert PVD, Heidelberg, Germany and flame annealed prior to the preparation of the SAMs. BP2 SAMs were prepared by following a procedure described elsewhere [50]. The samples were immersed into solutions of 1 mM BP2 in ethanol at 345 K for about 15 h. After rinsing and blowing dry with nitrogen, the samples were annealed in a sealed container under nitrogen atmosphere at 418 K for about 10 h. The annealing transforms the SAM structure that was obtained at room temperature into the highly ordered δ-phase [50], which is used in the experiments.

**STM.** Structural characterisation and patterning was done with a PicoPlus microscope (Molecular Imaging) including a bipotentiostat and PicoLITH software. The tips were fabricated by chemically etching a Pt/Ir (80:20, GoodFellow) wire in a 2 M KSCN/0.5 M KOH mixture applying an AC current. Subsequently, they were coated with polyethylene to minimize Faradaic currents. Typical tunneling parameters were in the range of 50 pA, 0.5 V for imaging in air, and 50 pA, 0.17–0.30 V for EC-STM.

**Patterning and deposition.** For patterning of the BP2 SAM and the subsequent Cu-UPD, the sample was mounted on a sample plate inside a custom-built EC-STM Teflon cell and positioned in the STM. After patterning under ambient atmosphere the electrochemical cell was filled without moving the sample. For Cu-UPD an aqueous solution of 50 mM CuSO_4_/50 mM H_2_SO_4_, and Pt and Cu wires serving as counter and reference electrodes were used. All potentials are referenced to Cu^2+^/Cu. Before filling in the electrolyte, the sample potential was set to +0.4 V. UPD was performed at potentials in the range of 0–300 mV, depending on the desired deposition rate.

**Generation of binary SAM**. The exchange of BP2 by AdSH was done in a 0.1 M KOH ethanol solution containing 1 mM AdSH. In a single voltammetric cycle BP2 was desorbed in the cathodic sweep and AdSH adsorbed during the anodic sweep. The scan rate was set to 0.1 V/s. The successful exchange was verified by a second cycle, which showed a cathodic shift in the desorption potential (see Figure S1 in Supporting Information File 1), in accordance with the lower stability of an AdSH SAM compared to a BP2 SAM [43]. It is noted that the smaller peak area of the AdSH peak arises from the lower packing density of the AdSH molecules compared to BP2. The thiol exchange experiments were performed by removing the sample holder from the STM after Cu-UPD, then replace the Cu electrolyte by the AdSH containing electrolyte and swap the Cu reference electrode for Pt. To find the submicrometer patterns again after remounting the sample in the STM, a custom-made base plate was used with indentations that allow for a reproducible repositioning of the sample. However, due to the limited precision a scanner with a larger range (100 × 100 μm^2^) was used, in contrast to the experiments involving only patterning and UPD, which were also possible with a small range scanner (1.5 × 1.5 μm^2^).

## Supporting Information

A sequence of two linear sweep voltammograms is presented, which show the anodic shift in the reductive desorption peak of the thiol upon replacement of BP2 by AdSH.

File 1 Further experimental data.
