# Supplementary material for: Nanoscale patterning of a self-assembled monolayer by modification of the molecule–substrate bond
Source: Beilstein J Nanotechnol. 2014 Mar 10;5:258–67. doi: 10.3762/bjnano.5.28 (PMC3999799; doi:10.3762/bjnano.5.28)
Supplement: File 1 — Further experimental data. [file Beilstein_J_Nanotechnol-05-258-s001.pdf]

Supporting Information

for

Nanoscale patterning of a self-assembled monolayer by modification of the molecule–substrate bond

Cai Shen[1,2] and Manfred Buck[*1]

Address: [1]EaStCHEM School of Chemistry, University of St Andrews, St Andrews KY16 9ST, United Kingdom and [2]present address: Ningbo Institute of Materials Technology & Engineering, Chinese Academy of Sciences, Ningbo, 315201, China.

Email: Manfred Buck* - mb45@st-andrews.ac.uk

*Corresponding author

Further experimental data

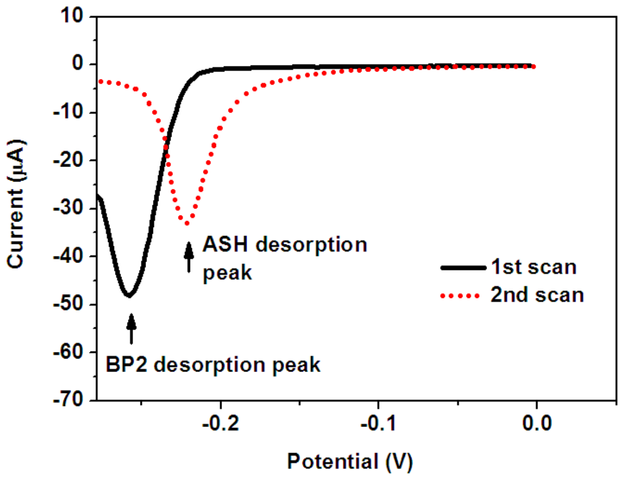

Figure S1: Cathodic sweeps showing the exchange of BP2 by AdSH. In the first scan, BP2 is desorbed from Au in the cathodic sweep, followed by the adsorption of AdSH during the anodic sweep. In the second scan, the desorption peak is shifted to the less cathodic potential of AdSH, thus demonstrating the quick backfilling of the Au areas by AdSH. Electrolyte: 1 mM AdSH in EtOH/KOH solution. Scan rate 0.1 V/s. Area of working electrode 0.47 cm^2. Potential is relative to a Pt pseudo reference electrode.
